# The tRNA Elbow in Structure, Recognition and Evolution

**DOI:** 10.3390/life6010003

**Published:** 2016-01-12

**Authors:** Jinwei Zhang, Adrian R. Ferré-D’Amaré

**Affiliations:** 1 Laboratory of Molecular Biology, National Institute of Diabetes and Digestive and Kidney Diseases, 50 South Drive, Bethesda, MD 20892, USA; 2 Laboratory of RNA Biophysics and Cellular Physiology, National Heart, Lung and Blood Institute, 50 South Drive, Bethesda, MD 20892, USA

**Keywords:** tRNA elbow, RNA structure, base stacking, ribosome, T-loop, convergent evolution

## Abstract

Prominent in the L-shaped three-dimensional structure of tRNAs is the “elbow” where their two orthogonal helical stacks meet. It has a conserved structure arising from the interaction of the terminal loops of the D- and T-stem-loops, and presents to solution a flat face of a tertiary base pair between the D- and T-loops. In addition to the ribosome, which interacts with the elbow in all three of its tRNA binding sites, several cellular RNAs and many proteins are known to recognize the elbow. At least three classes of non-coding RNAs, namely 23S rRNA, ribonuclease P, and the T-box riboswitches, recognize the tRNA elbow employing an identical structural motif consisting of two interdigitated T-loops. In contrast, structural solutions to tRNA-elbow recognition by proteins are varied. Some enzymes responsible for post-transcriptional tRNA modification even disrupt the elbow structure in order to access their substrate nucleotides. The evolutionary origin of the elbow is mysterious, but, because it does not explicitly participate in the flow of genetic information, it has been proposed to be a late innovation. Regardless, it is biologically essential. Even some viruses that hijack the cellular machinery using tRNA decoys have convergently evolved near-perfect mimics of the tRNA elbow.

## 1. Introduction

**Figure 1:**
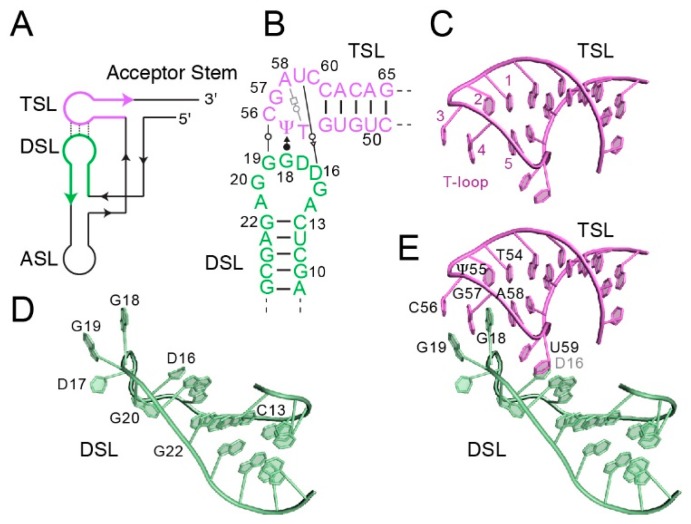
Structure of the tRNA elbow. (**A**) Schematic of the connectivity of tRNA. ASL, anticodon stem-loop. DSL, D-stem-loop. TSL, T-stem-loop. (**B**) Secondary structure of the yeast tRNA^Phe^ elbow. Non-canonical pairs between the D- and T-loops are depicted with Leontis-Westhof [29] symbols. Residue numbering reflects the tRNA convention. (**C**) Structure of the T-loop of yeast tRNA^Phe^ (PDB ID 1EHZ). Note the unstacked residue 56 corresponding to position 3 of the generalized T-loop, and the gap between residues 57 and 58, corresponding to positions 4 and 5 of the generalized T-loop. (**D**) Structure of the D-loop of yeast tRNA^Phe^. Dihydrouridines are located at residues 16 and 17. (**E**) Interaction of D- and T-loops forms the elbow. Note inter-loop base pairs between residues 19 and 56, and intercalation of D-loop residue 18 into the T-loop. Structure figures were prepared with PyMol [30].

A defining feature of the three-dimensional structure of transfer RNA [1,2] is the “elbow” where nucleotides from the D- and T-loops interact to give rise to the canonical L-shape of tRNAs (Figure 1A). From the viewpoint of the central dogma of molecular biology [3], the essential elements of a tRNA might appear to be its anticodon, which decodes the mRNA triplet code, and its acceptor (or CCA) terminus, which, when esterified to its cognate amino acid, supports mRNA-directed protein synthesis. This vantage, which emphasizes the flow of genetic information, ignores the fact that tRNAs are concrete molecular entities with highly conserved three-dimensional structures. Indeed, the overall architecture of tRNA is so fundamental to its biological functions that viruses that hijack the cellular machinery by mimicking tRNA have convergently evolved molecular architectures that replicate, in idiosyncratic manners, both the overall shape and dimensions of tRNA as well as those of constituent structural features, including the elbow [4,5,6,7]. Crystallographic analyses of translating ribosomes have demonstrated how precisely L-shaped elongator tRNAs fit in the interface of the two ribosomal subunits [8,9,10,11,12]. Crystal structures of tRNAs specifically bound to other RNAs or proteins show, in a number of cases, prominent interactions with the elbow region (Table 1). In this review, we survey tRNA structural biology from an “elbow-centric” perspective and suggest that the appearance of the tRNA elbow was a crucial event in the evolution of the modern translation machinery.

**Table 1:** Selected examples of recognition of the tRNA elbow.

Name	Type/Region of Polymer	Function	Mode of Interaction *	PDB Code	Ref.
Ribosome A site	23S RNA; helix 38 and others	Translation	H,V	4V6F	[13]
Ribosome P site	L5 protein and others	Translation	H,V	4V51	[10]
Ribosome E site	23S RNA; L1 stalk and others	Translation	S	4V4I	[11]
RNase P	RNA: J11/12–J12/11	tRNA modification: 5′ end maturation	S	3Q1Q	[14]
T-box riboswitch	RNA: Stem I distal region	Amino acid surveillance: tRNA binding, recognition of aminoacylation, and genetic switching	S	4LCK	[15]
LeuRS	protein	tRNA aminoacylation	H, V	2V0G	[16]
ValRS	protein	tRNA aminoacylation	H, V	1GAX	[17]
GatDE	protein	Aminoacyl-tRNA transamidation	unknown	2D6F	[18]
GatCAB	protein	Aminoacyl-tRNA transamidation	H, V	3AL0	[19]
RNase Z	protein	tRNA modification: 3′ end maturation	H, V	4GCW	[20]
DusC	protein	tRNA modification: reduction of D-loop U16/U20 to dihydrouridine	H, V	4YCP	[21]
TruB	protein	tRNA modification: pseudouridylation of T-loop U55 to Ψ	T-loop extraction	1K8W	[22]
ArcTgt	protein	tRNA modification: transglycosylation of D-loop G15 to PreQ_0_.	D-loop extraction	1J2B	[23]
CCA-adding enzyme	protein: tail domain	tRNA maturation; addition of 3′ CCA trinucleotide	H,V	1SZ1	[24]
CC-adding enzyme	protein: tail domain	tRNA maturation; addition of 3′ C nucleotides	H,V	3WFR	[25]
A-adding enzyme	protein	tRNA maturation; addition of 3′ A nucleotide	H,V	4X0B	[26]

* H: Hydrogen bonds; S: Stacking interaction; V: van der Waals interaction.

## 2. Anatomy of the tRNA Elbow

Transfer RNAs are subject to a remarkably diverse and conserved array of post-transcriptional modifications, and the two loops that form the elbow take their names, respectively, from dihydrouridine (D-loop) and ribothymidine (TΨC-loop, where T and Ψ denote ribothymidine and pseudouridine, respectively, or simply the T-loop). In elongator tRNAs, the D-loop is canonically comprised of ten residues, of which residues C13 and G22 (in yeast tRNA^Phe^) form the closing Watson-Crick pair (Figure 1B–E). The fourth and fifth residues of the D-loop (residues 16 and 17 in the conventional numbering scheme) are typically modified into dihydrouridines. The D-loop adopts an irregular structure from which the dihydrouridine at position 16 as well as two conserved guanine nucleobases (residues 18–19) are extruded, allowing all three to participate in tertiary interactions. The T-loop of tRNAs was the first described example of a widespread pentaloop RNA structural motif [27]. It is typically closed by a reverse Hoogsteen U•A pair between residues 1 and 5 of the motif. The intervening trinucleotide forms a U-turn such that residue 2 stacks on 1, residue 3 is unstacked, and a gap is present between residues 4 and 5 (Figure 1C). The latter gap and residue 3 are often involved in inter- and intra-molecular contacts such as intercalation and base-triple formation [28]. Indeed, in the tRNA elbow, the nucleotide at the third T-loop position (corresponding to residue 56 in the conventional tRNA numbering scheme) forms a Watson-Crick base pair with residue 19 from the D-loop, and D-loop residue 18 intercalates between the fourth and fifth nucleotides of the T-loop (Figure 1B,E). Overall, this results in mutual intercalation of D- and T-loop nucleobases such that residues A58, G18, G57, G19, and C56 form a continuous stack (Figure 1E). Other tertiary interactions that stabilize the elbow conformation include the formation of a Watson-Crick base pair between D-loop residue 15 and variable (V) loop residue 48, and a single-hydrogen bond pair between D-loop residue G18 and the universally conserved Ψ55 at the second position in the T-loop (Figure 1E). In addition to stabilizing the L-shape of tRNA, the elbow is characterized by presenting a flat hydrophobic surface to the solvent: the distal face of the tertiary Watson-Crick pair between residues G19 and C56. This unusual exposed base pair is exploited for tRNA elbow recognition by many proteins, RNAs and the ribosome.

## 3. Engagement of the tRNA Elbow by the Ribosome

**Figure 2:**
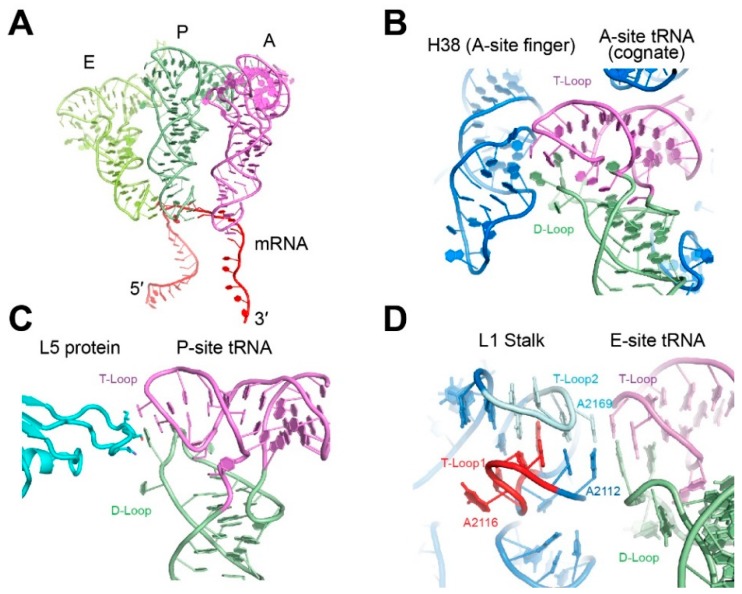
Interaction of the tRNA elbow with the ribosome. (**A**) Relative positions of the three classical state tRNAs from ribosome cocrystal structures. (PDB ID 4V6F). (**B**) Interaction of the tRNA elbow with helix 38 (the “A-site finger”). (PDB ID 4V6F) rRNA is in blue. (**C**) Interaction of the tRNA elbow with the L5 protein (cyan) in the P-site. (PDB ID 4V51). (**D**) Interaction of the tRNA elbow with the L1 stalk in the E-site. (PDB ID 4V4I). The two interdigitated T-loops of the L1 stalk are denoted T-Loop1 and T-Loop2 in the 5′ to 3′ direction.

**Figure 3:**
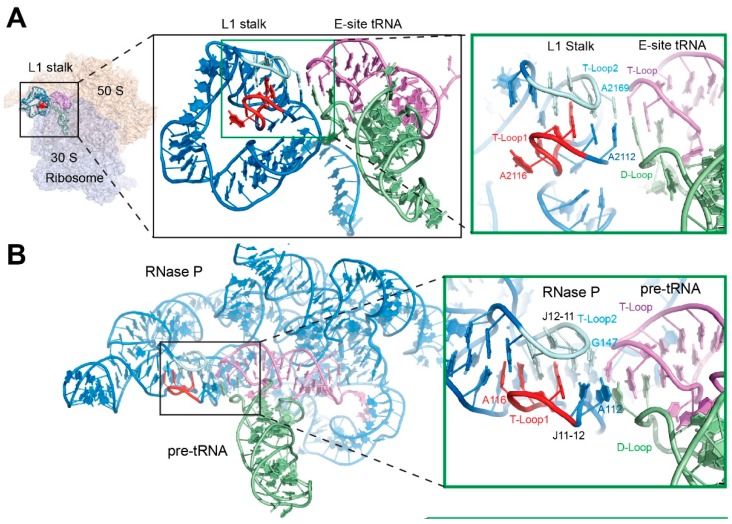

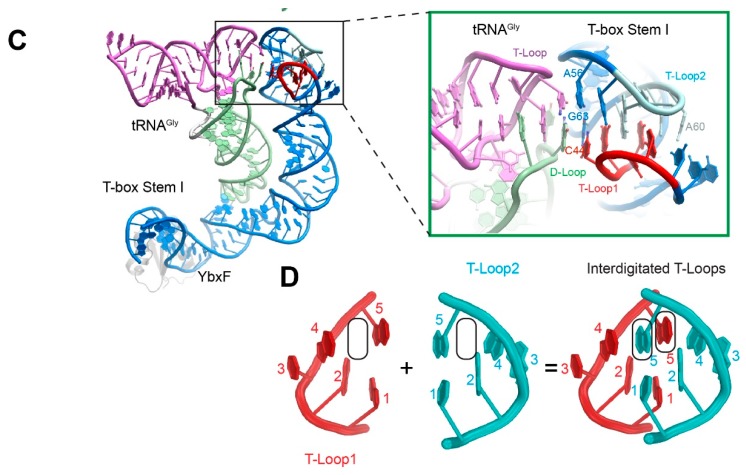
Recognition of the tRNA elbow by the interdigitated T-loop motif. (**A**) The 5′ and 3′ interdigitated pentaloops of the ribosomal L1 stalk are colored red and cyan, respectively. The tRNA D- and T-loops are pale green and violet, respectively. (PDB ID 4V4I). (**B**) Structure of RNase P holoenzyme bound to pre-tRNA. (PDB ID 3Q1Q). (**C**) Structure of a glycine-specific T-box Stem I domain bound to its cognate tRNA^Gly^. (PDB ID 4LCK). (**D**) Interdigitation of two head-to-tail pentanucleotide T-loops (red and cyan, respectively) forms a densely packed core structure. The five nucleotides that form each T-loop are numbered and the stacking gaps denoted by the rounded rectangles.

In modern biology, the *raison d’être* for most tRNAs is to enable protein synthesis, and they have thus evolved to have near-equivalent interactions with the ribosome. Elongator tRNAs transit through the three distinct sites on the ribosome, aminoacyl, peptidyl and exit sites (A, P, E; Figure 2A) at the interface of the small and the large ribosomal subunits. There, tRNAs are encased by both RNA and protein components of the ribosome. At all three sites, the tRNA elbow is recognized. Upon binding of a cognate tRNA to the A site, the entire anticodon stem loop (ASL) is first monitored by the rRNA, followed by inspection of the tRNA elbow by helix 38 of the 23S rRNA (A-site Finger, Figure 2B) [13]. The recognition of the expected tRNA elbow structure in the A site helps trigger the accommodation of cognate tRNA and thus contributes to tRNA proofreading. At the P site, the tRNA is in contact with many ribosomal protein tails, with protein L5 directly contacting the G19•C56 tertiary base pair of the tRNA elbow (Figure 2C) [10]. This L5-tRNA elbow interaction appears to contribute to the ribosomal grip of the peptidyl-tRNA. Structural destabilizations of the tRNA elbow dramatically slows down ribosomal translocation and exert strong effects on frame shifting [31,32,33]. After peptidyl transfer, the deacylated tRNA is moved to the E site where part of 23S rRNA forms a mobile element of the large ribosomal subunit termed the L1 stalk, which holds the tRNA by the elbow (Figure 2D and Figure 3A). By pivoting about its junction with the rest of the 23S rRNA, the L1 stalk moves to facilitate the ejection of tRNAs from the E site [11,34].

## 4. Recognition of the tRNA Elbow by Non-Coding RNAs

The sheer size of the ribosome provides an encompassing, closed environment for tRNA recognition and manipulation, within which a multitude of contacts, both from ribosomal proteins and rRNA, inspect, stabilize, deform, and translocate the tRNAs. Outside the ribosome, tRNAs are recognized by other, smaller non-coding RNAs in a more open structural context. The tRNA elbow is an essential feature by which some structured RNAs distinguish tRNAs from other RNAs, increase binding avidity and specificity through multivalent interactions, and function as “molecular rulers”.

Ribonuclease (RNase) P, the near-universal ribozyme [35] responsible for the endonucleolytic cleavage of pre-tRNA 5′ leader sequences, clamps on the pre-tRNA elbow and measures a defined distance along the TSL (T-stem-loop)-Acceptor Stem coaxial helical stack to locate the appropriate site of cleavage (Figure 3B) [14]. The T-box riboswitches, which are widespread in Gram-positive bacteria, monitor and maintain intracellular amino acid supplies [36,37]. They directly sense tRNA aminoacylation levels by forming a C-shaped structural wrapper around their cognate tRNAs (Figure 3C) [15,38]. They achieve this by exploiting the flexibility, which is afforded by their multi-domain structure, the precise placement of RNA structural motifs, and the construction of a steric sieving device that snugly fits the non-aminoacylated tRNA 3′ end [15,39,40]. The cotranscriptionally folded T-box RNA progressively engages the tRNA anticodon, the elbow, and the acceptor end, ultimately creating a metastable tRNA-mRNA complex that couples exquisite sensing of tRNA aminoacylation status to alternative RNA structure formation, thus enabling conditional genetic switching [40].

First suggested by bioinformatics and modeling analyses [41] and subsequently demonstrated by X-ray crystallographic structure determinations, 23S rRNA in the ribosomal E-site, RNase P and the T-box riboswitches have convergently adopted a common structural solution to recognizing the tRNA elbow. All three RNAs recognize the tRNA (or pre-tRNA) elbow, primarily through base stacking, employing a compact motif constructed by interdigitating a pair of T-loops (Figure 3A–D). The head-to-tail intermeshing of two pentanucleotide T-loops allows the fifth residue from each T-loop to occupy the stacking gap between residues 4 and 5 of the other T-loop, and to form a base-triple with its own residue 1 and its partner’s residue 2 (Figure 3D) [15,38]. The reciprocal intercalation that fills the stacking gaps and the formation of two central stacked base triples lead to formation of a stable and highly stacked core. This core then directs the placement of both intervening and flanking single-stranded regions and ultimately leads to the presentation of flat, stackable surfaces on both faces of the motif. The interdigitated T-loop motif constitutes a capable device for recognizing the characteristically flat tRNA elbow. The utilization of this structural motif by three independently evolved structured RNAs is likely a product of convergent evolution, because the orientation of the motif (that is, which of the two interdigitated T-loops of the motif is in contact with the tRNA elbow) differs between the three RNAs, and because the structural context of the motif within 23S rRNA, RNase P, and T-boxes has no resemblance. The recurrent use of this motif attests to its effectiveness and portability in tRNA recognition [15,41]. Presumably other examples of the interdigitated T-loops recognizing tRNA or tRNA-like structures will be discovered as transcriptomes are characterized structurally.

## 5. Diversity in Protein Recognition of the tRNA Elbow

In contrast to the convergent use of interdigitated T-loops by RNAs, the strategies used by proteins to recognize the tRNA elbow are diverse and variable. Consistent with the preferred modes for weakly polar interactions in proteins [42,43], tRNA-binding proteins disfavor parallel stacking of aromatic amino acid side-chains on the flat surface of the tRNA elbow. Instead, they use hydrogen bonding to nucleobase functional groups, 2′-OHs, and non-bridging phosphate oxygen atoms of elbow residues. Occasionally cation-π interactions are employed. Among the many proteins that bind tRNA, aminoacyl-tRNA synthetases (aaRSs), end-processing and maturation enzymes, and post-transcriptional tRNA modification enzymes illustrate diverse strategies of tRNA elbow recognition.

**Figure 4:**
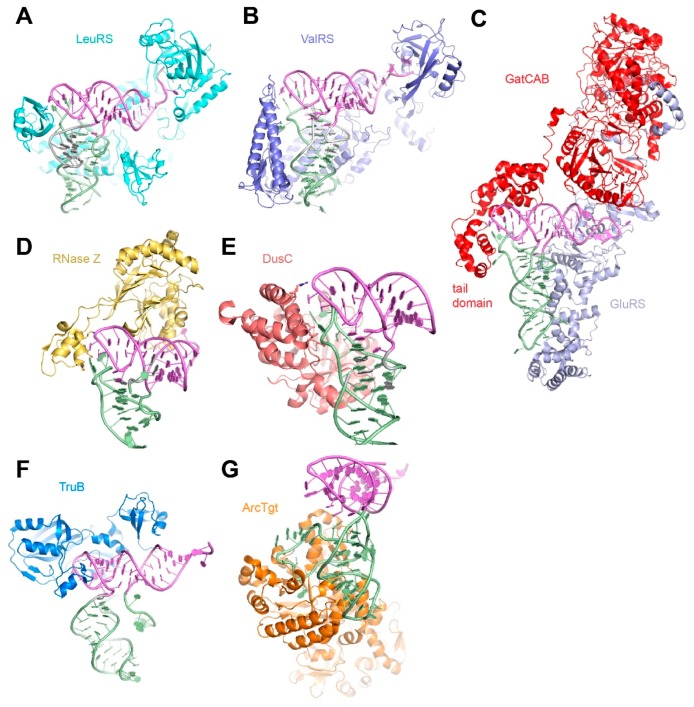
Recognition of the tRNA elbow by tRNA-binding proteins. (**A**) Structure of LeuRS recognizing its cognate tRNA^Leu^. (PDB ID 2V0G). (**B**) Structure of ValRS recognizing its cognate tRNA^Val^. (PDB ID 1GAX). (**C**) Structure of bacterial amidotransferase GatCAB complexed with GluRS recognizing its cognate tRNA^Gln^. (PDB ID 3AL0). (**D**) Structure of RNase Z recognizing pre-tRNA^Thr^. (PDB ID 4GCW). (**E**) Structure of the dihydrouridine synthetase DusC bound to tRNA^Trp^. (PDB ID 4YCP). (**F**) Model of pseudouridine synthase TruB bound to λ-form tRNA^Val^ based on its cocrystal structure in complex with a tRNA minihelix. (PDB ID 1K8W and 1J2B). (**G**) Structure of archaeosine transglycosylase ArcTGT bound to λ-form tRNA^Val^ (PDB ID 1J2B).

Arguably the most important tRNA-binding proteins, aaRSs catalyze the activation of amino acids by forming an aminoacyl-adenylate intermediate, and subsequently transfer the activated aminoacyl group to either the 2′- or 3′-OH of their cognate tRNAs. These enzymes accurately identify the correct tRNA and the correct amino acid before covalently linking them, in order to ensure that the genetic code is faithfully translated. Some also perform post-transfer proofreading [44]. Most aaRSs recognize their cognate tRNAs through direct interactions with the anticodon and acceptor stem regions (such as the discriminator base N73). A smaller number of aaRSs (such as LeuRS, ValRS) also interact with the tRNA elbow, which can potentially serve as additional tRNA identity elements in those cases (Figure 4A,B) [16,17]. In two fascinating examples, the archaeal and bacterial glutamine amidotransferases (the heterodimeric GatDE and heterotrimeric GatCAB, respectively), distinguish cognate substrate tRNA^Gln^ from noncognate tRNA^Glu^ in part by employing shape complementarity between their tail domains and the smaller D-loops at the elbow of tRNA^Gln^ (Figure 4C) [18,19]. Even when the elbow interactions do not contribute to tRNA specificity, they could increase overall functional affinity, or avidity of protein-tRNA association. This is exemplified by the helical domain of GatE, which binds the minor groove of the TSL near the elbow to provide an additional anchoring interaction but does not contribute to tRNA selectivity [18].

Upon endonucleolytic cleavage of their 5′ ends by the elbow-clamping RNase P, pre-tRNAs are also processed at their 3′ ends by either exonucleases or a conserved 3′ endonuclease, RNase Z in the case of CCA-less pre-tRNAs. Reminiscent of the RNase P ribozyme, this protein uses a polypeptide protrusion called the “flexible arm” to recognize the tRNA elbow [45] (Figure 4D). This interaction contributes ~100 fold in binding affinity [46,47]. After RNase Z cleavage, the tRNA 3′ CCA termini are added by the CCA-adding enzyme or by the sequential action of CC- and A-adding enzymes [24]. These template-independent nucleotidyltranferases use their tail domains to measure the distance from the tRNA elbow to place the proper tRNA termini in their active sites (Table 1) [25,26].

Besides tRNA end-cleaving and end-extending enzymes and aaRSs, numerous tRNA modification enzymes elaborate tRNA with a large, diverse set of chemical modifications that confer additional chemical and structural features. These posttranscriptional modifications perform a wide range of functions including altering the decoding capability of anticodons and fine-tuning tRNA structure and dynamics for optimal transit through the ribosome [48]. Some of these enzymes recognize the tRNA elbow to modify other tRNA regions, and others directly target the T- and D-loops for chemical modification. Some of these latter enzymes, such as tRNA dihydrouridine synthase DusC [21], can access their substrate region (D-loop) whilst maintaining contact to the tRNA elbow (Figure 4E). Other enzymes that must dig deeper into the elbow region, exemplified by the pseudouridine synthase TruB [22], and archaeosine tRNA-guanine transglycosylase ArcTgt [23], disrupt the elbow structure to access their substrate nucleotides (Figure 4F,G), leaving the tRNA in a splayed-open, λ-like shape. These tRNA structures, in which the D-loop is forced away from the T-loop, are suggestive of the structural plasticity and late evolution of the D-loop.

## 6. The tRNA Elbow in Evolution

It is generally believed that primordial tRNA-like molecules consisted only of the ASL and Acceptor Stem, which function in decoding of mRNA codons and esterification to an amino acid, respectively [49,50,51]. In this view, the tRNA elbow would have been a relatively late addition. Geometrically speaking, the existence of the elbow was likely driven by the need to bend the tRNA, in order to simultaneously juxtapose the anticodons and acceptor ends of immediately adjacent tRNAs in the P- and A-sites [41,52]. Conceivably, the bending of tRNA structure to enable codon-directed peptidyl transfer could be achieved by other types of “elbows” that don’t involve the presentation of a flat, hydrophobic surface.

Because of its many functions in modern biology, the evolution of the contemporary tRNA elbow could have been a watershed event that demarcated old and new modalities of tRNA structure and, consequently, its recognition. In an era that predates the tRNA elbow, protein and RNA machines that interfaced with proto-tRNAs would have had to interact with either the ASL or the Acceptor Stem. Consistent with this notion, most aaRSs, among the most ancient tRNA-binding proteins, predominantly recognize the non-elbow features of tRNA. A caveat to this idea is that aaRSs must effectively distinguish various tRNA subtypes including ones that have highly similar overall structures. The commonality of the elbow structure across different tRNAs makes it less useful for establishing an amino acid-specific tRNA-aaRS interaction. Nonetheless, recognition of the tRNA elbow can significantly increase binding avidity and also ensures that the tRNA in question conforms to the expected L shape. In contrast to aaRSs, tRNA-binding proteins that emerged after the watershed, as exemplified by the enzymes that catalyze many tRNA post-transcriptional modifications (particularly those in the D- and T-loops) and the aforementioned aminoacyl-tRNA transamidation enzymes, recognize the tRNA elbow.

Besides proteins, various cellular RNAs recognize the tRNA elbow. The ribosome L1 stalk, RNase P, and the T-box riboswitches even converged on the same structural motif, the interdigitated T-loops, to recognize the elbow. A number of metazoan mitochondrial tRNAs lack the tRNA elbow structure. Consistent with this, the mitochondrial ribosomal L1 region and RNase Ps also lack the sequences that would form the interdigitated T-loops [41,53,54,55,56]. Similarly, there are no known T-boxes that operate within mitochondria. These observations support the notion that the tRNA elbow co-evolved with the molecular entities that must interface with tRNA. Interestingly, some T-box riboswitches, such as the *ileS* T-boxes from *Actinomyces*, are truncated at the top of Stem I so that they lack the interdigitated T-loops canonically employed for tRNA elbow recognition [57]. Is it possible that these atypical T-boxes are so evolutionarily ancient that they predate the tRNA elbow?

The importance of the tRNA elbow to cellular physiology is further evidenced by the fact that a number of viral tRNA-like RNAs go to great lengths to mimic the tRNA elbow structure, despite having divergent topologies in other parts. The crystal structure of the Turnip Yellow Mosaic Virus (TYMV) tRNA-like element reveals that this RNA employs a D/T-loop association nearly identical to that of a canonical tRNA while having substantially different structural strategies (such as use of pseudoknotting) elsewhere [4]. Mimicry of tRNA structure, including the characteristic elbow, allows viruses to hijack many host tRNA-binding molecules, including the translation factor eEF1A, aaRSs, tRNA modification enzymes, RNase P, and even the ribosome [5]. Curiously, other cellular RNAs, including several long noncoding RNAs that only evolved fairly recently, also employ tRNA mimicry to recruit tRNA-processing enzymes like RNase P for their end maturation [58].

The tRNA elbow is likely a relatively late addition to cellular tRNA structure. Its widespread adoption and retention may be driven by its significant contribution to an improved fold of the tRNA, or to more effective transit through the ribosome. The tRNA elbow would have become fixed through the “principle of many users” [59], once multiple recognition events started relying on this novel molecular feature for recognition. The elbow-containing tRNA would then have become subject to further targeting and mimicry by newly evolved cellular systems and foreign systems such as RNA viruses and retroviruses.
